# The predictive value of triglyceride-glucose index on early neurological functional improvement in non-diabetic patients with acute ischemic stroke undergoing intravenous thrombolysis

**DOI:** 10.3389/fneur.2025.1626196

**Published:** 2025-09-22

**Authors:** Furong Li, Xiaowen Sui, Xin Pan, Jun Li, Yan Gao, Dandan Shi, Hongling Zhao, Dong Chen

**Affiliations:** 1 Stroke Center, Central Hospital of Dalian University of Technology (Dalian Municipal Central Hospital), Dalian, China; 2 Neurology Department, Central Hospital of Dalian University of Technology (Dalian Municipal Central Hospital), Dalian, China; 3 Neurosurgery Department, Central Hospital of Dalian University of Technology (Dalian Municipal Central Hospital), Dalian, China

**Keywords:** acute ischemic stroke, intravenous thrombolysis, early neurological improvement, triglyceride-glucose index, non-diabetic patients

## Abstract

**Objective:**

To explore the predictive role of the triglyceride-glucose index (TyG index) on the early neurological improvement in non-diabetic patients with acute ischemic stroke (AIS) undergoing alteplase intravenous thrombolysis (IV-rtPA).

**Methods:**

This study included 490 AIS patients without diabetes, whose time from onset to hospital time ≤3 h undergoing IV-rtPA in the Stroke Center of our hospital from September 2023 to September 2024 through the Stroke Emergency Map Management Platform in Dalian City. According to the National Institutes of Health Stroke Scale (NIHSS) score at 24 h after IVT, the patients were divided into early neurological improvement (ENI) group (*n* = 332) and non-ENI group (*n* = 158). General information, risk factors, experimental data and the location of cerebral infarction were collected. Intergroup analyses were conducted using univariate or multivariate logistic regression.

**Results:**

(1) In the ENI group, blood glucose (FBG), triglycerides (TG), TyG index, and baseline NIHSS score were significantly lower than those in the non-ENI group (*p* < 0.05). (2) Binary logistic regression analysis indicated that a TyG index ≤7.15 and a low baseline NIHSS score could predict early neurological improvement undergoing intravenous thrombolysis (IVT) in AIS patients. The area under the curve (AUC) values for the TyG index, baseline NIHSS score, and the combined variable (Y) in predicting ENI were 0.640, 0.641, and 0.721, respectively, with the combined variable (Y) exhibiting the highest AUC value.

**Conclusion:**

The TyG index, baseline NIHSS score, and the combined variable (Y) are predictors of early neurological improvement, with the combined variable (Y) exhibiting a higher predictive efficiency.

## 1 Introduction

Currently, intravenous thrombolysis remains the primary treatment option for acute ischemic stroke (AIS) within the therapeutic time window (1), yet some patients still experience a poor long-term prognosis. It is therefore crucial to investigate the risk factors and measurable biomarkers that influence the early neurological outcomes of AIS patients post-intravenous thrombolysis.

Insulin resistance (IR), recognized as the primary pathophysiological mediator of metabolic syndrome, is deemed a significant contributor to the onset and progression of atherosclerosis and cardiovascular and cerebrovascular diseases (2). Research (3) has indicated that elevated IR levels are linked to adverse neurological outcomes in patients with AIS. Analysis of data from 273,368 cases in the UK Biobank has revealed that the triglyceride-glucose index (TyG index) surpasses individual blood glucose and triglyceride levels in forecasting stroke incidence, suggesting that the TyG index is an effective biomarker for IR in predicting stroke outcomes and a novel surrogate marker for IR (4). Recent studies have proposed that the TyG index is correlated with atherosclerosis (5, 6), serves as an independent predictor of cardiovascular events, and is associated with poor prognoses in patients with cardiovascular diseases (7, 8). Nevertheless, there is a relative scarcity of studies examining the correlation between the TyG index and the prognosis of AIS patients. This study aims to investigate the predictive value of the TyG index for the early neurological function of non-diabetic AIS patients undergoing alteplase intravenous thrombolysis (IV-rtPA), thereby aiding clinicians in rapidly assessing the prognosis of AIS patients post-intravenous thrombolysis with alteplase and in creating personalized treatment strategies.

## 2 Materials and methods

### 2.1 Patient selection

**Figure 1:**
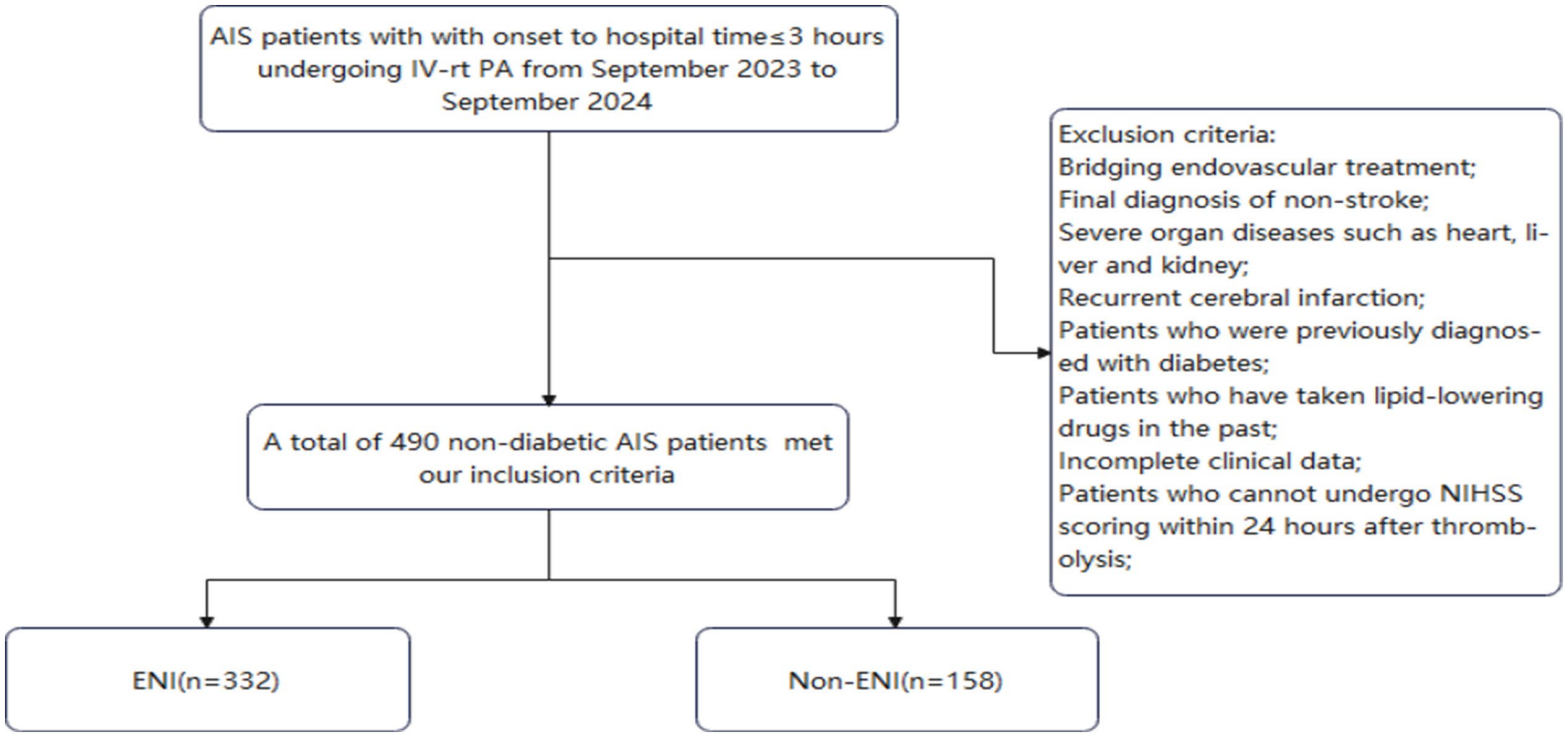
Flow of included patients through the trial.

This study conducted a retrospective analysis, included 490 non-diabetic patients with AIS who presented within 3 h of symptom onset and underwent IV-rtPA therapy in the Stroke Center of our hospital from September 2023 to September 2024 through the Stroke Emergency Map Management Platform in Dalian City. Inclusion criteria were as follows: meeting the Chinese Guidelines for Diagnosis and Treatment of Acute Ischemic Stroke 2023 and being first-time stroke cases, patients were eligible for IV-rtPA therapy, with successful completion of the procedure. Exclusion criteria included patients receiving bridging endovascular therapy; those diagnosed with non-stroke conditions; patients with severe cardiac, hepatic, renal, or other organ dysfunction; patients with recurrent AIS; diabetic patients; individuals who had previously taken statins; patients with incomplete clinical data; and those who failed neurological deficit assessment 24 h post-thrombolysis. According to the National Institutes of Health Stroke Scale (NIHSS) score after IVT, patients showing a decrease of ≥4 points compared to baseline or achieving complete recovery (i.e., NIHSS score of 0) were classified as the early neurologic improvement (ENI) group (9, 10), other patients were classified as the non-ENI group. The ENI group (*n* = 332): 94 males and 64 females, with an average age of 68.48 ± 12.38 years; the non-ENI group (*n* = 158): 240 males and 92 females, with an average age of 68.19 ± 12.51 years (Figure 1).

### 2.2 Data collection

Collect the general information of the patients (age, gender), Body Mass Index (BMI), cerebrovascular disease risk factors (hypertension, atrial fibrillation, smoking, drinking); systolic pressure (SBP), diastolic pressure (DBP) and NIHSS score before thrombolysis; time from onset to hospital arrival (min); and time from hospital arrival to IVT (min); The TOAST classification (11) for AIS was used for etiological classification of stroke [large-artery atherosclerosis (LAA), cardioembolism (CE), small-artery occlusion lacunar (SAO), stroke of other determined etiology (SOE), and stroke of undetermined etiology (SUE)]; the distribution of stroke (anterior circulation, posterior circulation, and both anterior and posterior circulation) and the occurrence of intracerebral hemorrhage transformation were assessed. The laboratory data included the white blood cell count and neutrophil count before thrombolysis; fasting blood glucose (FBG), total cholesterol (TC), triglycerides (TG), low-density lipoprotein (LDL), uric acid, and creatinine within 24 h after thrombolysis for analysis (instrument model: Siemens Healthineers ADVIA Chemistry XPT fully automated biochemical analysis system). TyG index = ln [TG (mg/dL) × FBG (mg/dL)/2] (12).

### 2.3 Statistical processing

SPSS 22.0 was used in statistical processing. Normally distributed measurement data were expressed as (^−^x ± s) and compared between groups via an independent sample t-test. Nonnormally distributed measurement data were described using M (interquartile range), and intergroup comparisons were performed using the Mann–Whitney U test. Enumeration data were presented as n (%) and compared through a chi-square test. Influencing factors were identified through binary or ordered multiclass logistic regression analysis. The predictive value of indicators was evaluated by plotting a receiver operating characteristic (ROC) curve. *p* < 0.05 was considered statistically significant.

## 3 Results

Based on the NIHSS score within 24 h, the 490 non-diabetic patients with AIS were divided into the ENI group (*n* = 332, 67.76%) and the non-ENI group (*n* = 158, 32.24%). The two groups exhibited no statistically significant differences in general patient data and vascular risk factors (*p* > 0.05), indicating comparability. In the ENI group, FBG, TG, TyG index and baseline NIHSS score were significantly lower than those in non-ENI group (*p* < 0.05), suggesting that the above factors are all influencing factors for early neurological improvement (Table 1).

**Table 1:** Comparison of risk factors between ENI and non-ENI groups.

Categories	ENI groups (*n* = 332)	Non-ENI groups (*n* = 158)	t/χ2	*p*
Age (year)	68.19 ± 12.51	68.48 ± 12.38	0.173	0.863
Gender (%)			4.038	0.054
Female	92 (27.71)	64 (40.51)		
BMI (kg/m^2^)	24.65 (23.23–25.9)	24.46 (22.9–26.48)	0.67	0.312
Hypertension (%)	220 (66.27)	106 (67.09)	0.016	0.898
Atrial fibrillation (%)	34 (10.24)	6 (3.80)	2.964	0.085
Smoking (%)	170 (51.20)	70 (44.30)	1.02	0.312
Alcohol consumption (%)	98 (29.52)	40 (25.32)	0.467	0.494
Time from onset to hospital arrival (min)	120.00 (60.0,180.0)	120.00 (60.0,150.0)	−0.972	0.331
Time from hospital arrival to IVT (min)	29.00 (24.0,36.3)	27.00 (21.0,36.0)	−1.907	0.057
Baseline blood pressure (mmHg)				
Systolic blood pressure	142.50 (126.0,159.0)	147.00 (126.0,160.0)	−1.075	0.282
Diastolic blood pressure	86.00 (76.0,90.0)	86.00 (82.0,90.0)	−1.614	0.107
FBG (mmol/L)	5.60 (0.22,0.43)	6.07 (5.43,7.63)	−2.432	0.015
TG (mmol/L)	6.07 (5.43,7.63)	6.07 (5.43,7.63)	−2.914	0.004
TyGindex	7.30 (6.86,7.66)	7.51 (7.21,7.94)	−3.549	0.000
TC (mmol/L)	4.70 ± 1.18	4.67 ± 1.35	−0.151	0.880
LDL (mmol/L)	3.00 ± 0.98	2.92 ± 1.00	−0.588	0.557
Uric acid (umol/L)	333.09 ± 95.38	334.11 ± 88.74	0.080	0.936
Creatinine (umol/L)	66.00 (55.1,78.8)	64.60 (56.1,79.3)	−0.148	0.883
White blood cell count (10^9^/L)	6.965 (5.7,8.7)	6.75 (5.9,8.8)	−0.324	0.746
Neutrophil count (10^9^/L)	4.29 (3.2,5.5)	4.42 (3.5,6.1)	−0.879	0.380
Etiology of stroke (%)				
LAA	158 (47.59)	40 (50.63)	0.198	0.656
CE	32 (9.64)	6 (3.80)	2.553	0.110
SAO	138 (41.57)	70 (44.30)	0.164	0.685
SOE	2 (0.60)	2 (1.27)	0.291	0.590
SUE	4 (1.20)	2 (1.27)	0.222	0.968
Baseline NIHSS score (points)	5.00 (4.0,6.0)	7.00 (5.0,10.0)	−6.698	0.000
Stroke distribution (%)				
Anterior circulation	220 (66.27)	112 (70.89)	0.523	0.469
Posterior circulation	92 (27.71)	42 (26.58)	0.034	0.853
Anterior and posterior circulation	20 (6.02)	2 (1.27)	2.826	0.093
Hemorrhagic transformation (%)	4 (1.20)	2 (1.27)	0.002	0.968

Binary Logistic regression analysis was conducted to identify the factors influencing early neurological improvement. Univariate and multivariate analyses were performed on the TyG index, TG, FBG and baseline NIHSS score. The results ultimately indicated that the TyG index and baseline NIHSS score were independent influencing factors for early neurological improvement in non-diabetic AIS patients. Specifically, the lower the TyG index and the lower the baseline NIHSS score, the better the early neurological improvement (Tables 2, 3).

**Table 2:** Univariate regression analysis of early neurological improvement.

Categories	B	S.E.	Wald	*p*	OR	95% C.I. for OR	
TyG index	0.615	0.227	7.361	0.007	1.850	1.186	2.884
FBG	0.004	0.003	1.745	0.187	1.004	0.998	1.010
TG	0.003	0.005	0.414	0.520	1.003	0.994	1.012
Baseline NIHSS score	−0.307	0.055	31.372	0.000	0.736	0.661	0.819

**Table 3:** Multivariate regression analysis of early neurological function improvement.

Categories	B	S.E.	Wald	*p*	OR	95% C.I. for OR	
Baseline NIHSS score	−0.318	0.056	31.714	0.000	0.728	0.652	0.813
TyG index	0.656	0.229	8.223	0.004	1.928	1.231	3.019
Constant	−1.872	1.705	1.205	0.272	0.154		

**Figure 2:**
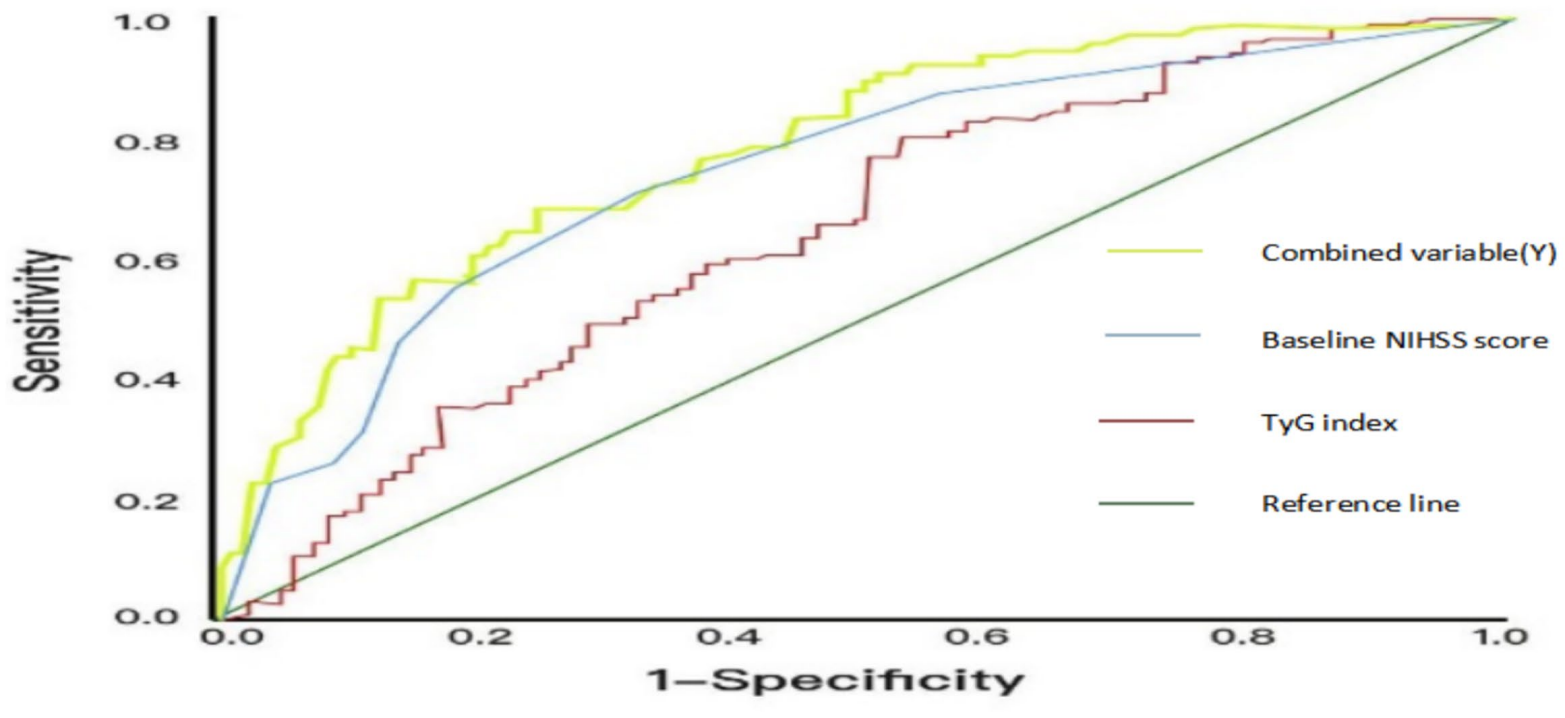
ROC curves of the predictive efficacy of TyG index, NIHSS score before thrombolysis and the combined variable (Y).

After determining the optimal cutoff value of 7.15 for the TyG index from Table 4, we used 7.15 as the threshold to convert the TyG index into a binary variable. AIS patients were then divided into a high TyG index group (TyG index > 7.15, *n* = 195) and a low TyG index group (TyG index ≤ 7.15, *n* = 295). The proportions of ENI in the high and low TyG index groups were 43.3 and 77.5%, respectively, showing statistically significant differences (*p* ≤ 0.001). Combined with Figure 2, the AUC values of the TyG index and baseline NIHSS score are 0.640 (*p* < 0.001) and 0.641 (*p* < 0.001), respectively. The AUC value of the combined variable (Y) is the largest at 0.721 (*p* < 0.001), indicating a higher predictive efficacy than that of a single indicator.

**Table 4:** Predictive value of TyG index and baseline NIHSS score for ENI.

Categories	AUC	*p*	95% CI	Sensitivity	Specificity	Cutoff value
TyG index	0.640	<0.001	(0.563,0.717)	0.801	0.468	7.15
Baseline NIHSS score	0.641	<0.001	(0.651,0.774)	0.814	0.489	
Combined variable (Y)	0.721	<0.001	(0.735,0.839)	0.821	0.509	

## 4 Discussion

Research on clinical prognostic predictors for AIS has consistently been a focal point in clinical studies, influenced by multiple factors. This paper compares various elements including patient gender, BMI index, vascular risk factors, baseline blood pressure, time from onset to intravenous thrombolysis, relevant laboratory indicators, etiological classification, cerebral infarction location, and cerebral hemorrhage transformation. Statistical differences were only observed in FBG, TG levels, TyG, and baseline NIHSS score.

In the pathological process of AIS, both metabolic disorder and inflammatory response in the body are closely related to the clinical prognosis of *Homo sapiens* (13). This study also focused on inflammatory markers, but primarily selected widely recognized cells that have damaging effects on ischemic brain tissue, namely leukocytes and neutrophils (14, 15). However, no statistically significant difference was observed between the ENI group and the non-ENI group in this research, which may be attributed to the limited scope of inflammatory markers examined. TyG is often closely associated with the body’s metabolic levels, particularly TG and blood glucose, and can indicate metabolic disorder. Furthermore, metabolic dysfunction can exacerbate the occurrence and progression of inflammatory response.

Currently, the pathogenesis of AIS resulting from IR is believed to involve primarily the following aspects: Firstly, reduced insulin activity diminishes glucose bioavailability, causing an imbalance in glucose and lipid metabolism. This imbalance leads to inflammation and oxidative stress. Excessive advanced glycation end products result in the proliferation of vascular smooth muscle cells, collagen deposition, vascular fibrosis, increased wall stiffness, endothelial cell dysfunction, and foam cell formation. Consequently, atherosclerosis progresses (16). Secondly, IR triggers the phosphorylation of insulin receptor substrates, a process mediated by insulin-like growth factor-1 and insulin-like growth factor-2. This, in turn, initiates C-reactive protein-mediated platelet activation, leading to an increase in platelet number and volume (17). Thirdly, IR contributes to cerebrovascular reserve (CVR) via the Bayliss effect (a myogenic mechanism), as well as through chemical, neural, and metabolic mechanisms, leading to hemodynamic disturbances. The CVR in individuals with IR is lower than that observed in healthy individuals (18–20). Fourthly, IR can amplify risk factors via inflammation and oxidative stress (21, 22), such as hypertension and diabetes, accelerate the progression of atherosclerosis, reduce the level of cerebral blood flow metabolism, and lead to the occurrence and recurrence of stroke. The cholinergic anti-inflammatory pathway (CAP) is a neuroimmune regulatory mechanism that plays a crucial role in immune balance and anti-inflammatory defense (23). Activating this pathway can alleviate obesity-induced inflammation and IR (24). The spleen and its sympathetic system constitute key components of the CAP pathway (23), with the spleen serving as a vital anti-inflammatory center in peripheral tissues. This suggests that spleen function may have played a significant role in early neurological improvement, though further exploration is required for confirmation.

In recent years, more and more studies have found that TyG index is closely related to ischemic stroke. However, a cohort study involving 5,014 healthy participants indicated that higher levels of the TyG index showed no significant correlation with cerebrovascular diseases (25). Nevertheless, some studies (26, 27) found that elevated TyG index was associated with stroke relapse in elderly AIS patients, and the TyG index might be a major risk factor for stroke among young adults in China. Cai et al. (28) found that TyG index can also be used as a potential risk stratification index for hospitalization and intensive care unit mortality in severe AIS patients. Lee et al. (29) found that a higher TyG index was significantly associated with a poor neurological prognosis in anterior circulation AIS patients receiving reperfusion therapy, and suggested that TyG index could help predict the short-term prognosis of AIS patients after reperfusion therapy. Zhang et al. (30) found that in patients receiving intravenous thrombolysis, a higher TyG index was associated with reduced early neurological improvement, suggesting that IR may play a role in the early neurological outcomes of AIS. Recently, a meta-analysis (31) indicates that elevated TyG index may increase cancer risk. Similarly, another meta-analysis (32) revealed significant IR resistance in cancer patients. Since IR may be a primary factor contributing to cancer-related metabolic dysfunction, this elevates risks of recurrence and mortality (32). Therefore, comprehensive tumor screening should be implemented for acute cerebral infarction patients to monitor Trophoblastic Tumor Syndrome (TTS). Additionally, attention should be paid to tumor development during long-term follow-up for secondary prevention of cerebral infarction patients.

Therefore, the choice of TyG index as a predictor of early neurological improvement in non-diabetic patients with AIS in this study is well justified. The results showed that the proportion of ENI was higher in the group with low TyG index than that in the group with high TyG index within 24 h of intravenous thrombolysis, proving that TyG index was an independent predictor of early neurological improvement in AIS patients after intravenous thrombolysis with alteplase. In addition, the NIHSS score before thrombolysis reflects the degree of neurological deficit and is also an important index for the clinical prognosis of patients, which is basically consistent with the results of previous studies (33). Therefore, the TyG index, when combined with the baseline NIHSS score, has a higher predictive value for early neurological improvement in non-diabetic patients with AIS than a single TyG index alone, which is basically consistent with previous studies. However, the combined AUC value of the two was only slightly higher than that of TyG index. The possible reasons are that the prognosis of acute cerebral infarction is affected by many factors, and the risk factors compared in this study are limited. In addition, the differences between pathogenesis, non-interventional risk factors and thrombolysis may also have an impact.

However, this study is retrospective and has certain limitations. Firstly, this experiment did not set up a healthy control group, and did not stratify and analyze the AIS patients subgroups with intravenous thrombolysis. Secondly, there are inevitable individual differences in the measurement of laboratory indicators, and only a single measurement of the TyG index after admission was evaluated, without a dynamic evaluation. The baseline NIHSS score, even if assessed by experienced neurologists, is subject to some subjective influences.

## 5 Conclusion

In conclusion, in patients with acute ischemic stroke treated with alteplase intravenously, the combination of TyG index and baseline NIHSS score had a higher predictive efficacy for early neurological improvement than the single TyG index. Large-scale, randomized controlled and multicenter prospective studies are needed for further demonstration in the future.

## Data Availability

The original contributions presented in the study are included in the article/supplementary material, further inquiries can be directed to the corresponding authors.
