# Correction: The predictive value of triglyceride-glucose index on early neurological functional improvement in non-diabetic patients with acute ischemic stroke undergoing intravenous thrombolysis

**DOI:** 10.3389/fneur.2026.1784552

**Published:** 2026-03-23

**Authors:** Furong Li, Xiaowen Sui, Xin Pan, Jun Li, Yan Gao, Dandan Shi, Hongling Zhao, Dong Chen

**Affiliations:** 1 Stroke Center, Central Hospital of Dalian University of Technology (Dalian Municipal Central Hospital), Dalian, China; 2 Neurology Department, Central Hospital of Dalian University of Technology (Dalian Municipal Central Hospital), Dalian, China; 3 Neurosurgery Department, Central Hospital of Dalian University of Technology (Dalian Municipal Central Hospital), Dalian, China

**Keywords:** acute ischemic stroke, intravenous thrombolysis, early neurological improvement, triglyceride-glucose index, non-diabetic patients

In the published article, the following DOI references appear incorrectly.

The DOI of reference 3 was erroneously written as 10.1055/a-1583-2912. It should be 10.1055/a-1648-7767.

The DOI of reference 9 was erroneously written as 10.1159/000506679. It should be 10.1159/0 00504 776.

The DOI of reference 10 was erroneously written as 10.1016/j.jocn.2018.06.044. It should be 10.1016/j.jocn.2018. 08.004.

The DOI of reference 13 was erroneously written as 10.1007/s42000-023-00435-9. It should be 10.1007/s42000-23-00438-6.

The DOI of reference for 16 was erroneously written as 10.1186/s12933-021-01399-z. It should be [10.1186/s12933-021-01391-7.

The DOI of reference 17 was erroneously written as 10.1186/s12933-022-01590-w. It should be [10.1186/s12933-022-01593-7.

The DOI of reference 19 was erroneously written as 10.1177/00033197211007759. It should be 10.1177/000 331972 11007719.

The DOI of reference 20 was erroneously written as 10.1186/s12933-022-01482-z. It should be 10.1186/s12933-022-01490-z.

The DOI of reference for 27 was erroneously written as 10.1186/s12944-022-01763-2. It should be 10.1186/s12944-023-01773-8.

The DOI of reference for 28 was erroneously written as 10.1186/s12933-023-01870-z. It should be 10.1186/s12933-023-01864-x.

The DOI of reference 29 was erroneously written as 10.1159/000519229. It should be 10.1159/000516950.

The DOI of reference 30 was erroneously written as 10.3390/jcm12093471. It should be 10.3390/jcm12103471.

The original version of this article has been updated.

